# LncRNA *SRA* mediates cell migration, invasion, and progression of ovarian cancer via NOTCH signaling and epithelial–mesenchymal transition

**DOI:** 10.1042/BSR20210565

**Published:** 2021-09-06

**Authors:** Lee Kyung Kim, Sun-Ae Park, Yoolhee Yang, Young Tae Kim, Tae-Hwe Heo, Hee Jung Kim

**Affiliations:** 1 Laboratory of Pharmacoimmunology, Integrated Research Institute of Pharmaceutical Sciences and BK21 FOUR Team for Advanced Program for SmartPharma Leaders, College of Pharmacy, The Catholic University of Korea, 43 Jibong-ro, Bucheon-si, Gyeonggi-do 14662, South Korea; 2 Institute of Women's Life Medical Science, Division of Gynecologic Oncology, Department of Obstetrics and Gynecology, Yonsei University College of Medicine, Seoul 03722, South Korea; 3 Department of Plastic Surgery, Samsung Medical Center, Sungkyunkwan University School of Medicine, Seoul 06351, South Korea

**Keywords:** epithelial-mesenchymal transition, invasion, migration, NOTCH, ovarian cancer, SRA

## Abstract

Long non-coding RNA (lncRNA) is a newly identified regulator of tumor formation and tumor progression. The function and expression of lncRNAs remain to be fully elucidated, but recent studies have begun to address their importance in human health and disease. The lncRNA, SRA, known as steroid receptor activator, acts as an important modulator of gynecological cancer, and its expression may affect biological functions including proliferation, apoptosis, steroid formation, and muscle development. However, it is still not well known whether SRA is involved in the regulation of ovarian cancer. The present study investigated the molecular function and association between SRA expression and clinicopathological factors. In ovarian cancer cell lines, SRA knockdown and overexpression regulated cell migration, proliferation, and invasion. Both *in vivo* and *in vitro* experiments using knockdown and overexpression showed that SRA potently regulated epithelial–mesenchymal transition (EMT) and NOTCH pathway components.

Further, clinical data confirmed that SRA was a significant predictor of overall survival (OS) and progression-free survival and patients with ovarian cancer exhibiting high expression of SRA exhibited higher recurrence rates than patients with low SRA expression. In conclusion, the present study indicates that SRA has clinical significance as its expression can predict the prognosis of ovarian cancer patients. High expression of the lncRNA SRA is strongly correlated with recurrence-free survival of ovarian cancer patients.

## Introduction

The most prevalent types of malignant tumors among women are cervical, breast, uterine, and ovarian cancer. Ovarian cancer is associated with malignant tumor mortality in women worldwide. According to the GLOBOCAN series reported by the International Cancer Institute, female cancer has a relatively poor prognosis with a 32.5% mortality rate [1]. Ovarian cancer is one of the most typical gynecological malignancies, with 21750 newly diagnosed cases each year, leading to high morbidity and mortality. The 5-year survival rates for ovarian cancer stages I and II range from 80 to 95% and the diagnosis rate at more advanced stages is less than 30% [2]. Due to the lack of effective treatment for recurrent cases, most patients relapse within 2 years and the mortality rate is the highest among gynecological malignant tumors [3,4]. Approximately 98% of all transcripts in the human genome represent RNAs that do not encode proteins [5]. These non-coding RNAs (ncRNAs) were previously considered transcriptional noise; however, there is growing evidence that they play an important role in most cell processes, including differentiation, cell proliferation, self-destruction, immunity, and metabolism [6]. Accumulating evidence has shown the involvement of long non-coding RNAs (lncRNAs) in pathological processes, especially tumorigenesis [7]. Dysregulated lncRNAs are usually observed in tumor tissues. They are capable of mediating malignant phenotypes of tumor cells and thus influence the progression and metastasis of tumors [8]. Vital function of lncRNAs in ovarian cancer have been extensively studied [9,10]. In 1999, the steroid receptor RNA activator (SRA) was first identified and it was found that lncRNA SRA may increase the activity of steroid receptors [11]. Since then, studies investigating the effects of lncRNA SRA on gene transcription control have increased. According to these studies, lncRNA SRA can control a variety of important cell functions, such as migration, proliferation, and cell invasion. lncRNA SRA is also associated with the onset of cardiovascular disease [12]. In addition, lncRNA SRA acts as a novel transcriptional co-activator. The lncRNA SRA is an RNA transcript that controls eukaryotic gene expression and plays an important role in eukaryotic development, reproduction, metabolism, and disease [13]. SRA has been associated with malignant tumor progression, but its role in ovarian cancer is not clear. Our previous studies have shown that lncRNA SRA expression in cervical cancer cell lines is related to the progression of malignant tumors [14]. Although, the biological function and clinical relevance of SRA in the progression of ovarian cancer has not been confirmed. The present study investigated the functional role of SRA in the progression of ovarian cancer. The results show that SRA regulates the growth, invasion, and migration of ovarian cancer cells through NOTCH signaling and epithelial–mesenchymal transition (EMT). The present study suggests that SRA is a promising prognostic factor and target for ovarian cancer treatment.

## Materials and methods

### Patients and tissue samples

Women who underwent surgery at Yonsei Severance Hospital, Yonsei University, Seoul, Korea from 2012 to 2018 were enrolled. The present study was carried out in accordance with the principles of the Declaration of Helsinki and the ethical guidelines of the Ethics Committee of Yonsei Severance Hospital. All clinical information was collected from medical records. Tissue samples of newly diagnosed stage I–IV ovarian cancer (International Federation of Gynecology and Obstetrics (FIGO) stage) were evaluated blindly without details of prior diagnosis. Additionally, the control group consisted of 63 normal ovarian tissue samples retrieved from women near menopause during hysterectomy with two-sided fallopian ovarian resection due to benign uterine disease including vigilant smooth myoma. The present study was approved by the Ethics Committee of Yonsei Severance Hospital (ethic code: 4-2012-0363) and informed consent was obtained from all patients. All samples were frozen in liquid nitrogen and stored at −80°C until use.

### Cell lines

The human epithelial ovarian cancer cell line SKOV3 was purchased from the Korean Cell Line Bank (KCLB, Seoul, South Korea) and the A2780 cell line was purchased from the European Collection of Authenticated Cell Cultures (Sigma–Aldrich, ECACC, St. Louis, MO, U.S.A.). Ovarian cancer cell lines OVCA429, OVCA433, HOSE, and TOV112D were provided by the Korean Gynecological Cancer Bank through the Biomedical Technology Development Program of the Ministry of Science, Information and Communication Technology and Future Planning (MSIP), Korea. OVCAR3, SKOV3, and A2780 cells were cultured in RPMI-1640 medium (Gibco, Gaithersburg, MD, U.S.A.). OVCA429, OVCA433, and TOV112D cells were cultured in Dulbecco’s modified Eagle’s medium. The HOSE cell line was cultured in ovarian epithelial cell medium (ScienCell, OEpiCM, Carlsbad, CA, U.S.A.). All culture media were supplemented with 10% (vol/vol) fetal bovine serum, 1% antibiotic–antimycotic and 1% penicillin/streptomycin. Cells were cultured in an environment maintained at 37°C in a humid atmosphere of 5% CO_2_ and 95% air. The culture medium was changed with fresh medium every 2–3 days and the cells were used at passages between 5 and 10.

### Quantitative real-time polymerase chain reaction

RNA was extracted from patient tissues and cultured cells using TRIzol reagent (Bioline, London, U.K.). Whole total RNA was returned to cDNA using a reverser reagent kit (Bioline) following the manufacturer’s instructions. Real-time PCR analysis (qRT-PCR) was performed using the SYBR Green Real-Time PCR Kit (Bioline). The settings for SRA amplification were as follows: initial denaturation at 95°C for 3 min, 40 cycles of denaturation at 95°C for 15 s, annealing at 60°C for 1 min, elongation at 60°C for 1 min, and final elongation at 72°C for 5 min. qRT-PCR was performed using the ABI StepOnePlus Real-Time PCR System (Applied Biosystems, Foster City, CA, U.S.A.). The results were normalized with the expression of U6. The relative change in mRNA expression was calculated using the 2^−ΔΔ*C*_T_^ method. All qRT-PCR experiments were repeated at least three times.The SRA primers used are shown in Supplementary Table S1.

### Small interfering RNA transfection

Knockdown of SRA in ovarian cancer cell lines was performed using small interfering RNAs (siRNAs). Negative control siNC and targeting siSRA were obtained from Genolution Pharmaceuticals Inc. (Genolution Pharmaceuticals Inc, Seoul, South Korea). Transfections were performed using G-fectin (Genolution Pharmaceuticals Inc) according to the manufacturer’s instructions. siRNA-transfected cells were subjected to *in vitro* analysis 48 h after transfection. This experiment was repeated at least three times. The siSRA target sequence is shown in Supplementary Table S1.

### Plasmid construction and generation of stable cell lines

The human full-length SRA cDNA was cloned and inserted into the pLenti6/V5-D-TOPO vector (4950-00, Invitrogen, CA, U.S.A.). Using the ViraPower Lentiviral Expression System (Invitrogen, CA, U.S.A.) according to the manufacturer’s instructions. The plasmid was transfected in 293FT cells for packaging and the resulting lentivirus was used to infect the desired cell line. SRA stably transfected cells were selected in media containing blasticidin (Invitrogen). The sequence map of the plasmid pLenti6/V5-D-TOPO and the overexpression fusion gene SRA have been described in previous studies [15].

### Cell proliferation assay

Cell proliferation was determined using the Cell Proliferation Kit-8 (Dojindo, Tokyo, Japan). Cells (5 × 10^4^ cells/well) were seeded into six-well plates in 2 ml of culture medium. After incubating overnight to allow cell adhesion and recovery, the cells were transfected with 30 nM siNC or siSRA for 0, 24, 48, 72, and 96 h. An aliquot (200 μl/well) cell counting kit-8 (CCK-8) solution was added to each well, followed by incubation for at 1 h at 37°C. Analysis of overexpression of cells was performed in the same manner except for siSRA transfection. An automatic microplate reader determined the optical density (OD) at 450 nm to calculate the number of surviving cells in each well. The analysis was performed three times.

### Matrigel invasion assay

Analysis was performed according to the BD Matrigel chamber protocol (pore size: 8 mm, BD Biosciences, U.S.A.). siSRA transfected cells were inoculated into six-well plates at 1 × 10^6^, transfected with siRNA 30 μM for 48 h and then 5 × 10^5^ cells were counted and inoculated into the upper chamber in serum-free medium and complete medium was added to the lower chamber.

As for the overexpression of cells, 5 × 10^5^ cells were counted in the same manner and inoculated in the upper chamber with serum-free medium and complete medium was added to the lower chamber. The invasion chamber was incubated for 48 h in an incubator set at 37°C and 5% CO_2_. After 48 h, the non-invaded cells from the top of the chamber were removed with a cotton swab. To identify invading cells under the filter, stained cells were stained using a staining reagent (Diff Quik, Sysmes, Kobe, Japan) and counted using NIH ImageJ.

### Wound-healing migration assay

Cell migration was assessed using a wound-healing assay. Approximately 5 × 10^5^ cells were seeded into six-well plates with culture medium and grown to 90% confluence in complete medium. The serum-containing medium was removed and the cells were serum-depleted for 24 h. When the cell density reached 100%, an artificial wound was generated with a sterile 200-μl pipette tip on the cell monolayer in six-well plates. After scratching, the wells were washed with phosphate-buffered saline (PBS) and filled with serum-free medium. Using a microscope, cell migration to the wound was captured at 0, 24, and 48 h. The width of the scratch was analyzed using NIH ImageJ software and calculated as a percentage of the closed scratch width (width at 0 h/width at 48 h). The results were normalized to control cells. The migrated cells were counted in ten fields under a 20× objective lens. The original magnification was 200×. The experiment was performed three times.

### Western blotting analysis

Cells were lysed and protein was extracted with RIPA buffer (Thermo Fisher Scientific Inc., MA, U.S.A.). Concentration of protein was measured using the Pierce BCA Protein Assay Kit (Thermo Fisher Scientific Inc.). Protein samples were boiled with 5× sample buffer, further resolved in 10% SDS/polyacrylamide gel and then electrophoretically transferred to polyvinylidene difluoride (PVDF) membranes (Millipore, Billerica, MA, U.S.A.). After being blocked with 5% skim milk in 1× Tris-buffered saline containing 0.1% Tween 20 (pH 7.6) for 1 h at room temperature, the membrane was stirred continuously and incubated with the following primary antibodies: N-cadherin (1:1000, 4061s, Cell Signaling, MA, U.S.A.), E-cadherin (1:1000, 3192s, Cell Signaling), β-catenin (1:1000, 9562s, Cell Signaling), Vimentin (1:1000, 3932s, Cell Signaling), Snail (1:1000, 3879s, Cell Signaling), NOTCH1 (1:1000, 3608s, Cell Signaling), NCID (1:1000, 4147s, Cell Signaling), P300 (1:1000, 86377s, Cell Signaling), HES1 (1:1000, 11988s, Cell Signaling), and β-actin (1:1000, 8457s, Cell Signaling) overnight at 4°C. Next the membranes were further incubated with secondary antibody Goat anti-Rabbit IgG (H+L) Secondary Antibody, HRP (1:3000, #65-6120, Invitrogen) to detect the immunoreactivity of the proteins. The Western blotting membranes were scanned using Bio-Rad ChemiDoc image system to visualize protein bands.

### Xenografts in mice

All animal experimental procedures were performed in aseptic conditions under constant temperature and humidity in accordance with Yonsei Medical University Protocol (IACUC NO: 2017-0205). BALB/c female mice (*n*=20, 5–6 weeks old, Orient Bio, Seongnam, South Korea) were caged in individually vented cages in groups of five animals with free access to food and water. OVCA433 cells pretreated with SRA overexpression and control vector were transplanted (1.0 × 10^6^ cells/flank, xenograft *n*=10) into the left dorsal scapula via subcutaneous injection. Tumor volume was estimated twice a week using calipers. Tumor volume were calculated using a simplified equation to estimate the rotational ellipsoid (length × width^2^ × 0.5). Each tumor was harvested approximately 25–30 days after treatment. Magnetic resonance imaging (MRI) was conducted using the Brucker Biospec 94/24 USR (9.4T) Small Animal Scanner (35-mm-diameter cage, Brut BioSpin MRI, Ettlingen, Germany). A custom cradle was used to hold each mouse during the MRI process. At the beginning of each imaging session, T2-weighted images were obtained using Quick Acquisition. These images were used to verify that the animal was in the correct position inside the magnetic bore. A mixture of 1.5% isofluoride at a 0.7 l/min flow rate and 1:1 O_2_/N_2_O was used as the anesthetic during MRI. Breathing was monitored using an air pillow.

The mouse’s temperature was kept within acceptable limits using circulating warm water. All animal experiments were conducted in the animal housing facilities at the ABMRC of Yonsei Medical University. All mice were killed with carbon dioxide release devices.

### Hematoxylin and Eosin staining

The tumor tissue was collected, fixed for 24 h with 4% paraformaldehyde, washed with PBS, and embedded in paraffin. Following the standard procedure, the 2-µm-section was stained with Hematoxylin and Eosin (SAMYOUNG, Gyeonggi-do, South Korea).

### Statistical analyses

Statistical analysis was performed using SPSS v 24.0 (IBM Corp., Armonk, NY, U.S.A.) for Windows software to analyze the data (SPSS Inc., Chicago, Illinois, U.S.A.). Pearson’s χ^2^ test, Student’s *t* tests, and Fisher’s exact test were used to evaluate the relationship between SRA expression and clinical pathological characteristics. To evaluate the model’s performance in terms of its ability to discriminate, the x^2^ value of the log rank test was used in a receiver operating characteristic (ROC) analysis. Overall survival (OS) was analyzed using the Kaplan–Meier method. The log-rank test was used to estimate differences between groups. The stepwise Cox proportional risk model was used in the multivariate survival analysis of important variables in a single random analysis. Statistical tests were considered two-sided and* P*-values 0.05 were considered statistically significant.

## Results

### The expression of SRA in ovarian cancer was high and correlated with poor prognosis

**Figure 1:**
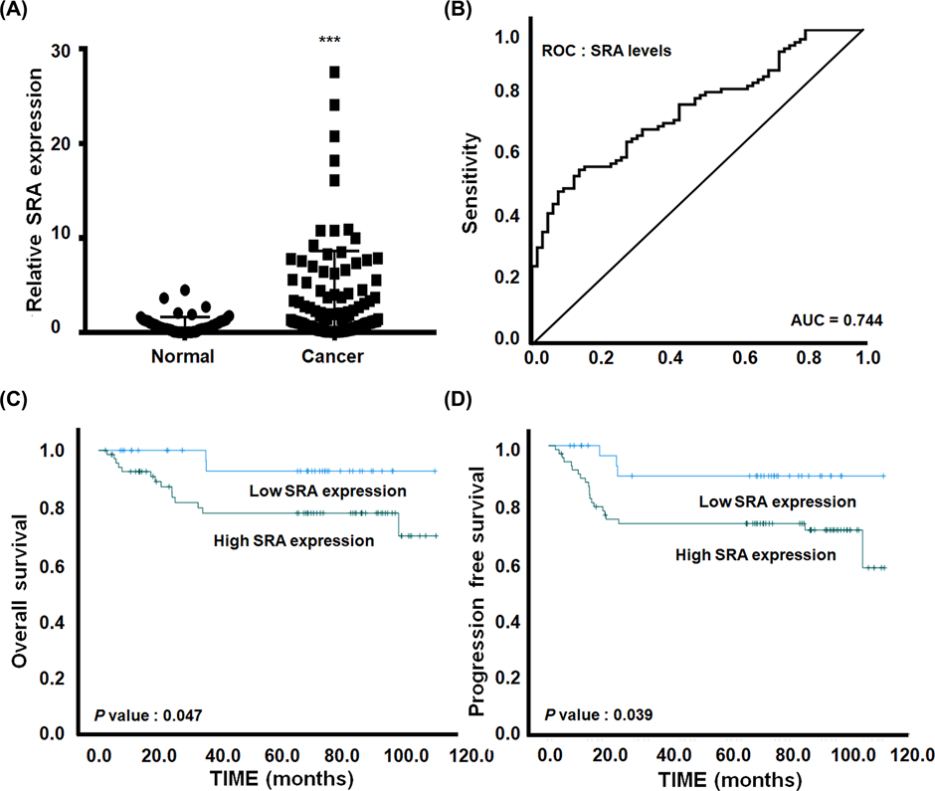
The clinical significance of SRA expression in ovarian cancer tissue (**A**) SRA expression is significantly higher in ovarian cancer tissues (*n*=101) than in non-cancerous tissues (*n*=63). SRA expression was determined using qRT-PCR and is expressed relative to the control value. Data are expressed as mean ± SD. ***P<0.001 vs. non-tumor control. (**B**) ROC curve for prognostic predictions stratifying patients by SRA levels. The AUC is indicated in the plots. (**C**) Kaplan–Meier curves for OS and (**D**) progression-free survival of ovarian cancer patients with different expression levels of SRA.

SRA expression was evaluated in ovarian cancer tissue (*n*=101) and in corresponding normal tissue (*n*=63) to determine whether SRA expression was related to the clinical pathological characteristics of ovarian cancer. SRA expression in ovarian cancer tissues was more than 4.52-times that in non-cancerous tissues (*P*=0.00002; Figure 1A). The predicted area under the curve (AUC) in the risk model for SRA data was 0.744 (*P*=0.000000145; Figure 1B). The characteristics of patients with high SRA expression (*n*=66) and low SRA expression (*n*=35) were compared (Supplementary Table S2). Kaplan–Meier survival analysis demonstrated that ovarian cancer patients with low SRA levels exhibited longer OS and progression-free survival than those with high SRA levels (*P*=0.047 and 0.039, respectively, Figure 1C,D). Additionally, univariate and multivariate analyses using the Cox proportional hazard model showed that SRA expression was a significant predictor for OS and recurrence survival (OS: multivariate HR = 5.106 (1.047–24.913), *P*=0.044; recurrence: univariate hazard ratio [HR] = 9.062 (2.982–27.536), *P*=0.0001, multivariate HR = 7.631 (2.258–25.784), *P*=0.001;) (Table 1). Both univariate and multivariate proportional hazard analyses showed that recurrence was an independent prognostic factor for OS. Multivariate Cox regression analysis for progression-free survival revealed that high SRA expression was an independent predictor of lymph node metastasis (univariate HR = 3.362 [1.538–7.351], *P*=0.001, multivariate HR = 2.543 (1.083–5.973), *P*=0.032) (Table 2).

**Table 1:** Univariate and multivariate analyses of various factors for OS

	OS			
	Univariate analysis		Multivariate analysis	
	HR (95% CI)	*P*	HR (95% CI)	*P*
SRA expression	4.016 (0.908–17.751)	0.067	5.106 (1.047–24.913)	0.044
Age, years (continuous)	1.014 (0.968–1.062)	0.562	0.980 (0.913–1.051)	0.572
FIGO stage	1.063 (0.706–1.598)	0.771	1.000 (0.643–1.555)	1
Grade	1.753 (0.721–4.260)	0.216	2.370 (0.824–6.819)	0.109
Cell type	0.722 (0.425–1.226)	0.228	0.852 (0.462–1.572)	0.608
Lymph node metastasis	1.912 (0.710–5.151)	0.2	1.149 (0.350–3.768)	0.819
Recurrence	9.062 (2.982–27.536)	0.0001	7.631 (2.258–25.784)	0.001
Menopause	6.237 (0.818–47.536)	0.077	8.879 (0.970–81.279)	0.053

**Table 2:** Univariate and multivariate analyses of various factors for progression-free survival

	Progression-free survival			
	Univariate analysis		Multivariate analysis	
	HR (95% CI)	*P*	HR (95% CI)	*P*
SRA expression	1.231 (0.562–2.694)	0.603	1.424 (0.635–3.190)	0.391
Age, years (continuous)	0.988 (0.957–1.020)	0.454	0.976 (0.934–1.020)	0.283
FIGO stage	1.243 (0.926–1.668)	0.148	1.157 (0.827–1.619)	0.394
Grade	1.003 (0.567–1.775)	0.991	1.119 (0.610–2.053)	0.716
Cell type	0.633 (0.397–1.008)	0.054	0.725 (0.455–1.154)	0.175
Lymph node metastasis	3.362 (1.538–7.351)	0.002	2.543 (1.083–5.973)	0.032
Menopause	1.011 (0.450–2.274)	0,979	1.034 (0.365–2.925)	0.95

### SRA expression increased in ovarian cancer cell lines and correlated with cell proliferation

**Figure 2:**
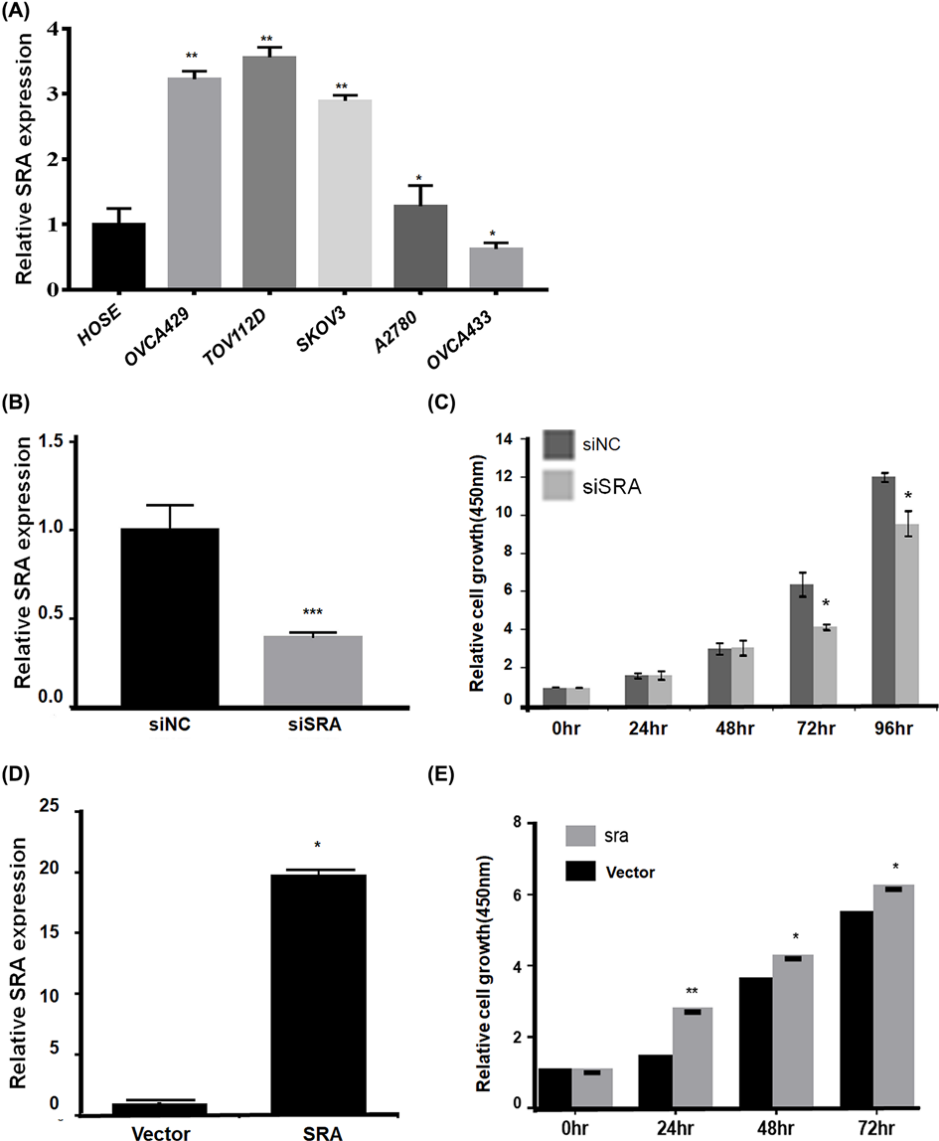
SRA expression and regulation of cell proliferation in ovarian cancer cell lines (**A**) The expression of SRA was significantly higher in some ovarian cancer cell lines (OVCA429, TOV112D, SKOV3, A2780, OVCA433) than in normal control cells (HOSE). *P<0.05, **P<0.01 vs. siNC. (**B**) OVCA429 cells were transfected with SRA-specific siRNA and negative-control siRNA and knockdown efficiency was determined by qRT-PCR analysis. qRT-PCR was performed in triplicate. ***P<0.001 vs. siNC. (**C**) The proliferation of OVCA429 cells transfected with siSRA and negative control siRNA was determined using the CCK-8 assay. Bars indicate mean ± SD of three independent experiments. *P<0.05 vs. siNC. (**D**) Overexpression of SRA in OVCA433 cells was determined by qRT-PCR analysis. qRT-PCR was performed triplicate. *P<0.05 vs. vector. (**E**) Cell proliferation was analyzed using CCK-8 assays. Bars indicate the mean ± SD of three independent experiments. *P<0.05, **P<0.01 vs. OVCA433, Vector cells. Abbreviation: SD, standard deviation.

SRA mRNA expression was evaluated in several ovarian cancer cell lines (Figure 2A). As shown in Figure 2A, TOV112D, OVCA429, SKOV3, and A2780 cells expressed higher levels of SRA than the control (HOSE) cells, but OVCA433 cells expressed lower levels of SRA than did the control (HOSE). Next, the effects of SRA on ovarian cancer cells were examined. To this end, SRA expression was knocked down by transfecting siSRA in OVCA429 cells and SRA was overexpressed in OVCA433 cells (Figure 2B,D). siSRA and SRA-overexpressing cells also exhibited differences in cell proliferation. siSRA showed inhibition of cell proliferation compared with siNC, while SRA overexpression showed enhanced cell proliferation compared with that of the control vector (Figure 2C,E).

### SRA knockdown and overexpression regulated the invasion and migration of ovarian cancer cells

**Figure 3:**
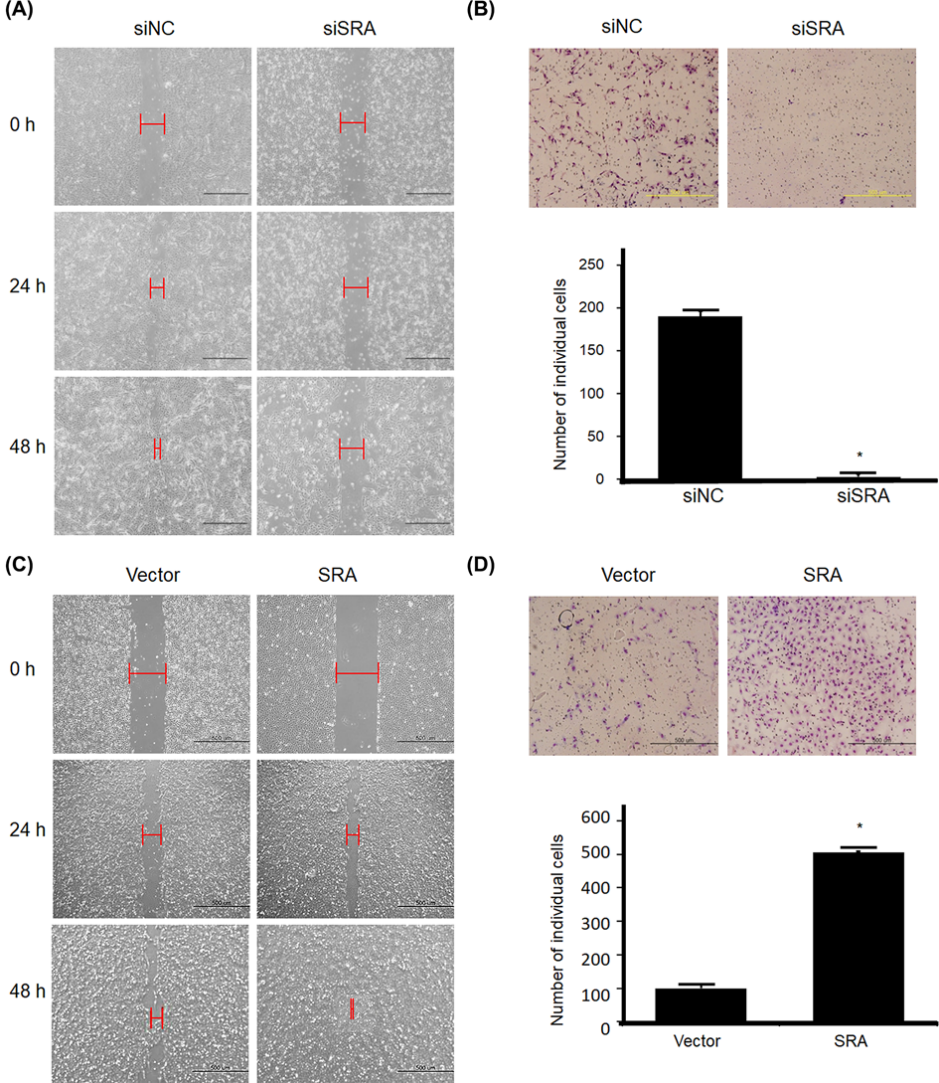
Knockdown and overexpression of SRA regulates the migration and invasion of OVCA429 and OVCA433 cells (**A**,**C**) A wound-healing assay was used to determine migration in siSRA-transfected OVCA429 and SRA-overexpression OVCA433 cells (×100). (**B**,**D**) Matrigel invasion assay was used to determine invasion after 48 h. Each assay was performed in triplicate. Data are mean ± SD. *P<0.05 vs. siNC and Vector.

We next evaluated whether SRA affected the invasion and migration of ovarian cancer cells. Cell invasion was evaluated after 48 h using the Matrigel invasion assay. SRA-knockdown OVCA429 cells showed a significant decrease in wound healing (Figure 3A). However, SRA overexpression in OVCA433 cells significantly enhanced wound healing (Figure 3C). In addition, the invasiveness of these cells was compared with that of control cells. SRA-knockdown cells exhibited reduced invasiveness (Figure 3B) and SRA-overexpression cells exhibited enhanced invasiveness (Figure 3D). Uncontrolled cell proliferation is a common biological feature in all tumors, but a major pathophysiological feature of malignant tumors is their ability to penetrate natural tissue barriers. The present findings suggested that SRA expression was related to cancer invasion and metastasis.

### SRA regulated the expression of EMT- and NOTCH-associated genes in ovarian cancer cells

**Figure 4:**
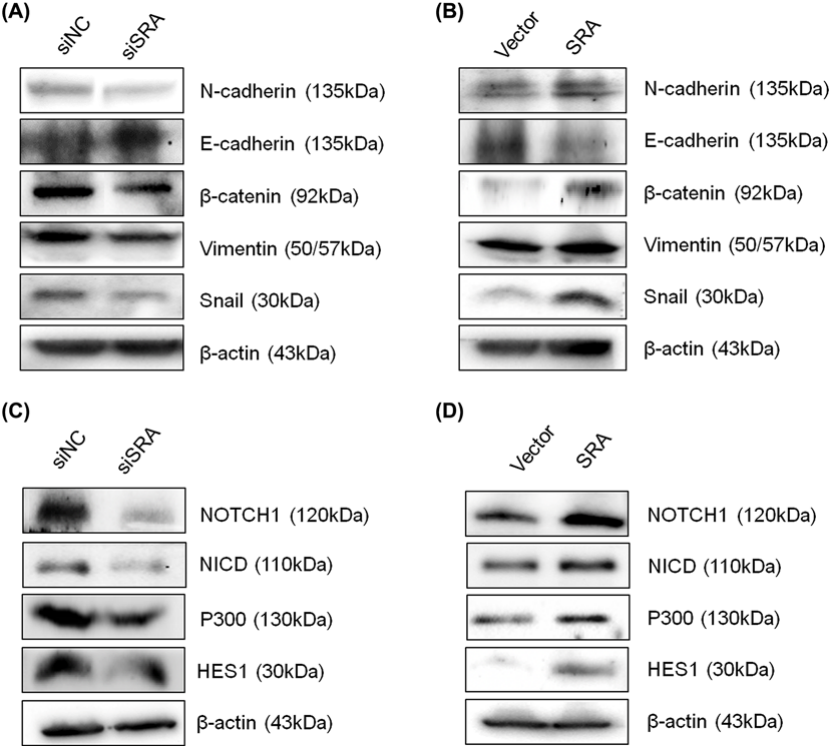
SRA regulates NOTCH and EMT signaling pathways Protein lysates were obtained 48 h after siSRA transfection of OVCA429 cell SRA-overexpression of OVCA433 cells. EMT (**A**,**B**) and NOTCH (**C**,**D**) related gene expression were analyzed by Western blotting.

Because EMT is important for cell migration and invasion, the identification of factors related to EMT could have clinical implications [16,17]. Therefore, the relationship between SRA and EMT was investigated. To this end, EMT-related markers were assessed by Western blotting (Figure 4A,B) after SRA knockdown in OVCA429 cells and after SRA overexpression in OVCA433 cells. SRA knockdown increased the expression of E-cadherin and decreased the expression of β-catenin, N-cadherin, Snail, and Vimentin. Conversely, SRA overexpression decreased the expression of E-cadherin and increased the expression of β-catenin, N-cadherin, Snail, and Vimentin. These results indicated that the dysregulation of EMT-related genes may be involved SRA-mediated effects on ovarian cancer cell migration and invasion. The NOTCH signaling pathway plays an important role in cell proliferation, differentiation, and cell death [18]. Therefore, the relationship between SRA and NOTCH signaling was investigated and NOTCH-related markers were estimated using Western blotting (Figure 4C,D). SRA knockdown in OVCA429 cells decreased NOTCH, NICD, P300, and HES1 expression, whereas SRA overexpression in OVCA433 cells increased NOTCH, NICD, P300, and HES1 expression. These results implicated that the dysregulation of NOTCH genes by SRA in ovarian cancer cell migration and invasion.

### SRA regulates tumor growth in a xenograft nude mouse model

**Figure 5:**
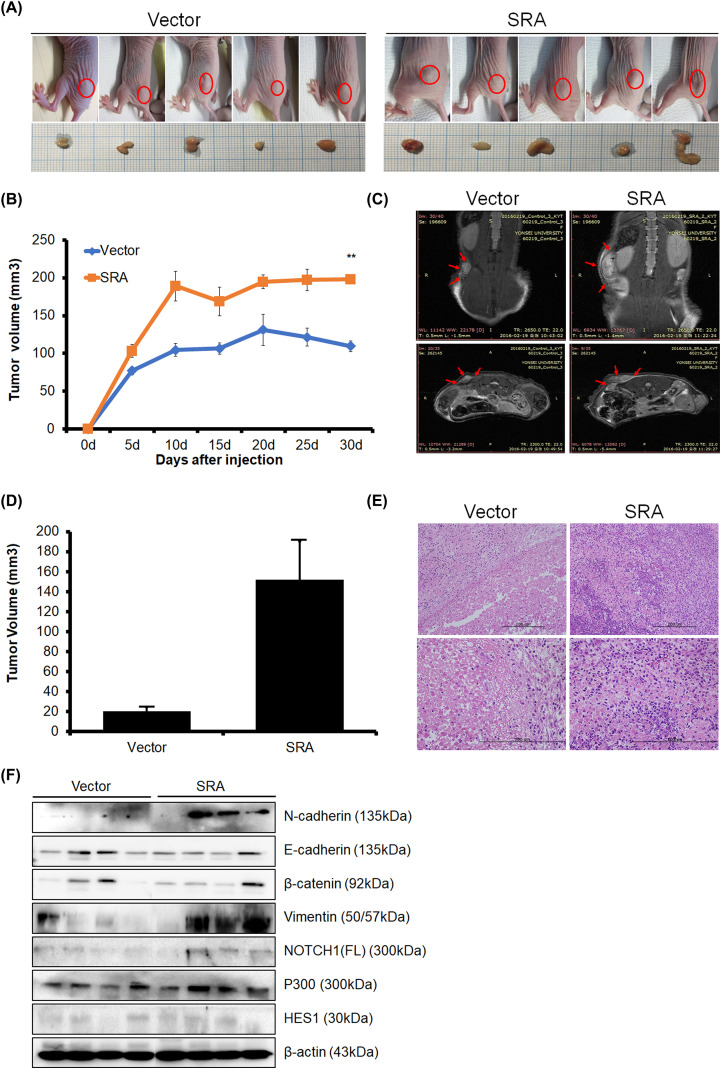
Effect of SRA on tumor growth *in vivo* SRA-overexpression OVCA433 cells were injected subcutaneously into the right dorsal scapula area of nude mice. (**A**) Representative gross images of tumor masses from all mice in each group. (**B**) Tumor volume was calculated every 5 days. Data are reported as mean ± SE (*n*=5). **P<0.001 vs. control. (**C**) MRI images. (**D**) Tumor sizes in the experimental groups according to MRI. Data are mean ± SE (*n*=5), vs. vector. (**E**) Hematoxylin and Eosin (H&E) staining of SRA-overexpression OVCA433 cells (×200). (**F**) The expression of EMT and NOTCH signaling was measured by Western blotting in xenograft model tumor tissues.

To explore whether SRA could affects tumor growth *in vivo*, OVCA433 cells, in which SRA was overexpressed, were implanted as xenografts in a nude mouse model. The results showed that the xenografts of the SRA-overexpression group had a larger tumor volume on day 25 than that of the vector xenografts (Figure 5A). The tumor volumes in each group are shown in Figure 5B. Tumor size and activity were further evaluated using MRI (Figure 5C). The tumor size of mice transplanted with SRA-overexpression OVCA433 cells was larger than that of mice transplanted with control empty vector cells (Figure 5D). Histological examination by Hematoxylin and Eosin tissue staining revealed that the SRA-overexpression xenograft had larger nucleoli and irregular nuclear membranes compared with those of the vector xenograft (Figure 5E). Utilizing tissues obtained from xenograft mice, EMT, and NOTCH-related markers were evaluated by Western blotting (Figure 5F).

Compared with the vector group, the expression of N-cadherin and Vimentin increased in the SRA group, and the expression of NOTCH1 and P300 slightly increased in the SRA group. In addition, SRA overexpression showed a slightly decreased expression of E-cadherin compared with the vector group. There were no differences in the expression of β-catenin and HES1. These findings suggested that SRA regulated tumor growth *in vivo*, further supporting the hypothesis that SRA is involved in the malignant transformation of ovarian cancer cells.

## Discussion

The role of lncRNA SRA has been extensively investigated in a variety of physiological and pathological processes, including breast cancer [19], the post-pubertal mammary gland [20], in myogenic differentiation [21], and hepatic steatosis [22]. However, the molecular mechanisms of lncRNA SRA involved in tumor progression and transitions are not fully understood. The expression of lncRNA SRA in ovarian cancer patients is significantly up-regulated, but its function and molecular mechanism in ovarian cancer are still unclear. Ovarian cancer is a fatal disease and EMT is recognized as making a significant contribution to its aggressive behavior [23]. EMT is a complex process that resolves incompletely and its treatment is clinically difficult to manage. Accordingly, it is important to identify important molecules that regulate EMT [24]. EMT involves the alteration of the cellular phenotype and several transcription factors have been identified that are involved in the regulation of EMT-related gene expression [25]. NOTCH signaling pathway plays an important role in the development and progression of human cancers and is critically involved in many cellular processes including cell proliferation, survival, apoptosis, migration, invasion, angiogenesis, and metastasis [26]. It is well known that the EMT process is stimulated and regulated by several signaling pathways, including transforming growth factor β, Hedgehog, Wnt, and NOTCH signaling [27]. New evidence indicates that the NOTCH signaling pathway plays an important role in the regulation of EMT, leading to tumor metastasis and invasion [26,28] and that NOTCH signaling inhibits EMT, thereby limiting the growth, invasion, and metastasis of gastric cancer [29]. In many cell types, only a few genes are commonly regulated by NOTCH signaling, while other target genes depend on the cell type or cell context [30]. In addition to directly regulating cancer-related genes, a cross-talk occurs with other oncogenic signaling pathways such as PI3K-Akt [31], NF-kB [32], and WNT signaling [33]. To better comprehend the direct role of SRA in carcinogenesis, EMT and NOTCH signaling were targeted with SRA knockdown and overexpression. The current study investigated the molecular function of SRA expression in ovarian cancer cell lines. The findings revealed that SRA regulates the growth, migration, and invasion of ovarian cancer cells. The loss of E-cadherin is thought to be an crucial event in EMT and N-cadherin diminishes the intercellular association between two adjacent endothelial cells, causing cancer cells to migrate [34]. Additionally, β-catenin moves and weakens the associated mesenchymal phenotype. Improvements in the expression of transcription factors, such as Snail, are associated with loss of attachment between cells [35]. Vimentin is a major constituent of the cytoskeleton of mesenchymal cells and its up-regulation is induced by EMT [36,37]. It has long been known that NOTCH signaling contributes to tumor progression (invasion, EMT, metastasis, and angiogenesis) and p300 is an important NOTCH co-activator [28]. This study evaluated whether EMT and NOTCH signaling pathways are compromised upon SRA overexpression and knockdown. There was an apparent association between increased expression of EMT- and NOTCH-related genes, P300 and HES1 in SRA-overexpressed OVCA433 cells. Conversely, SRA knockdown decreased EMT- and NOTCH-related gene expression in OVCA429 cells. Thus, SRA may contribute to ovarian cancer cell phenotypes via the activation of EMT and NOTCH signaling. Additionally, most patients with ovarian cancer have a high recurrence rate and those who relapse are often insensitive to chemotherapy and are usually incurable [38]. Therefore, it is critical to be able to predict patients at risk of disease relapse in advance; this will help to adopt more effective personalized treatment strategies with new drugs and targeted therapies to delay cancer recurrence and improve quality of life [39]. Improving the prognostication of patients with ovarian cancer requires firm predictions of recurrence and progression. Our findings correlated clinical data from 101 ovarian cancer patients and results from *in vivo* and *in vitro* experiments to support SRA as a predictable biomarkers for tumor recurrence. These findings suggested that SRA could potentially represent a novel biomarkers and therapeutic targets for ovarian cancer. In summary, we found that SRA expression was high in ovarian cancer tissues. Enhanced tissue SRA expression was positively correlated with clinicopathological parameters in *in vivo* and *in vitro* ovarian cancer cells. These results support the use of SRA to predict disease recurrence in patients with ovarian cancer. Additionally, SRA may also be a potential therapeutic target given its mechanistic role in promoting tumor invasion and cell proliferation by modulating EMT and NOTCH signaling pathways.

## Supplementary Material

Supplementary Figures S1-S2 and Tables S1-S2

## Data Availability

All data generated or analyzed during the present study are included in this published article.

## Abbreviations

AUC: area under the curve
EMT: epithelial–mesenchymal transition
lncRNA: long non-coding RNA
MRI: magnetic resonance imaging
OS: overall survival
PBS: phosphate-buffered saline
qRT-PCR: real-time PCR analysis
siRNA: small interfering RNA
SRA: steroid receptor activator
