# Supplementary material for: LncRNA SRA mediates cell migration, invasion, and progression of ovarian cancer via NOTCH signaling and epithelial–mesenchymal transition
Source: Biosci Rep. 2021 Sep 6;41(9):BSR20210565. doi: 10.1042/BSR20210565 (PMC8421593; doi:10.1042/BSR20210565)
Supplement: Supplementary Figures S1-S2 and Tables S1-S2 [file BSR-2021-0565_supp.pdf]

Table S1. SRA sequence

	Sequence 5' - 3'	
qRT-PCR sequence	Forward	CTCCCTTCTTACCACCACCA
	Reverse	TGCAGATACACAGGGAGCAG
siRNA sequence	CTCCCTTCTTACCACCACCA	

Table S2. Patient characteristics

Factor	n (%)	SRA expression		P-value[a]
		Low	High	
Age (mean±SD)	101	51.97±12.76	52.28±10.30	0.413
Stage				0.067
I	43	9	34	
II	7	4	3	
III	28	11	17	
IV	23	11	12	
Grade				0.086
I	7	3	4	
II	44	10	34	
III	50	22	28	
Lymphnode metastasis				0.255
Yes	47	19	28	
No	54	16	38	
Menopause				0.996
Yes	75	26	49	
No	26	9	17	
Recur				0.523
Yes	30	9	21	
No	71	26	45	
cell type				0.132
Serous	70	23	47	
Mucinous	9	2	7	
Endometrioid	13	4	9	
Clear	3	1	2	
Other	6	5	1	

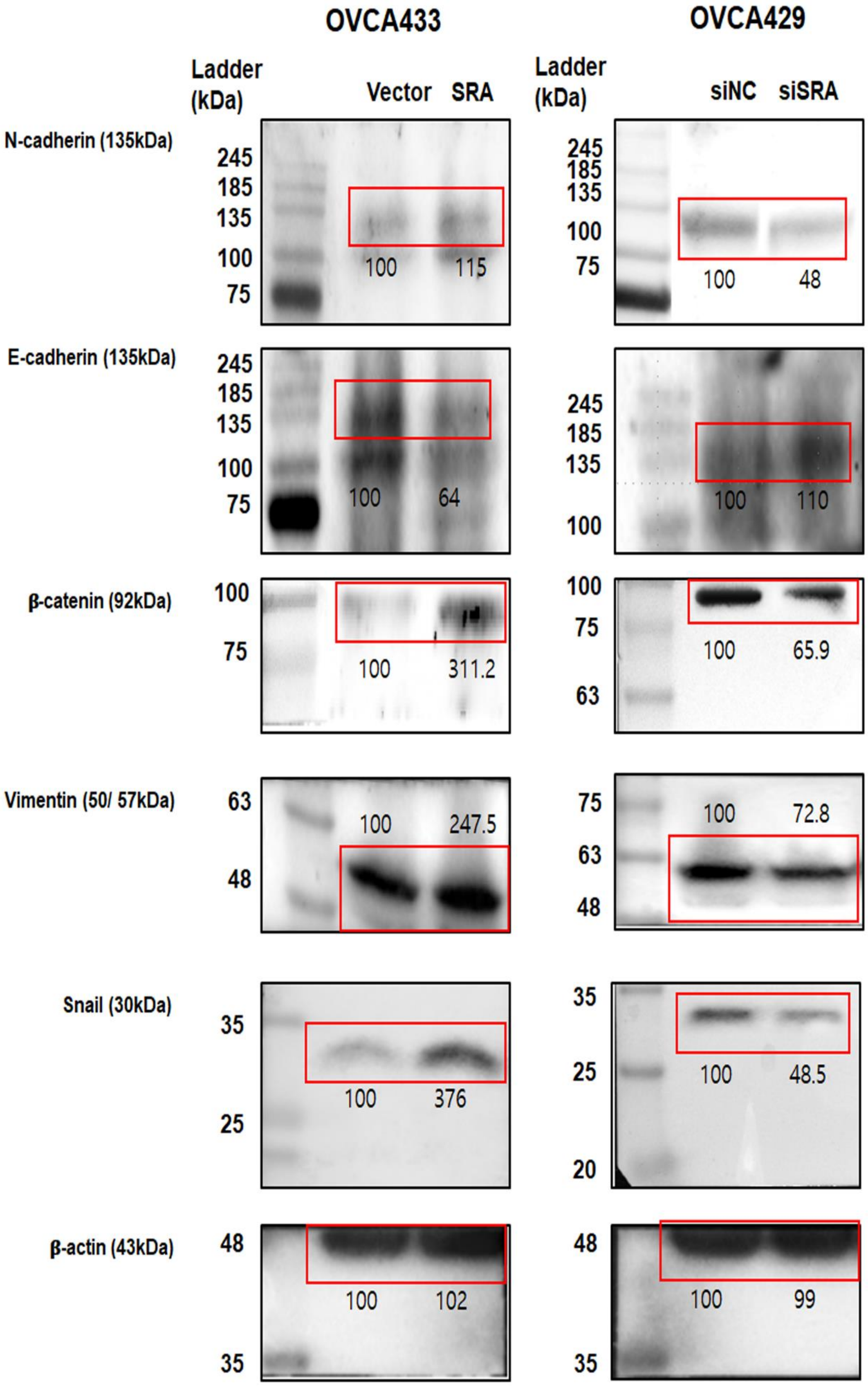
OVCA433
OVCA429
Ladder (kDa)
Vector
SRA
siNC
siSRA
N-cadherin (135kDa)
245
185
135
100
75
100
115
100
48
E-cadherin (135kDa)
100
64
100
110
β-catenin (92kDa)
100
311.2
100
65.9
63
Vimentin (50/ 57kDa)
63
48
100
247.5
75
100
72.8
Snail (30kDa)
35
25
100
376
100
48.5
20
β-actin (43kDa)
48
35
100
102
100
99

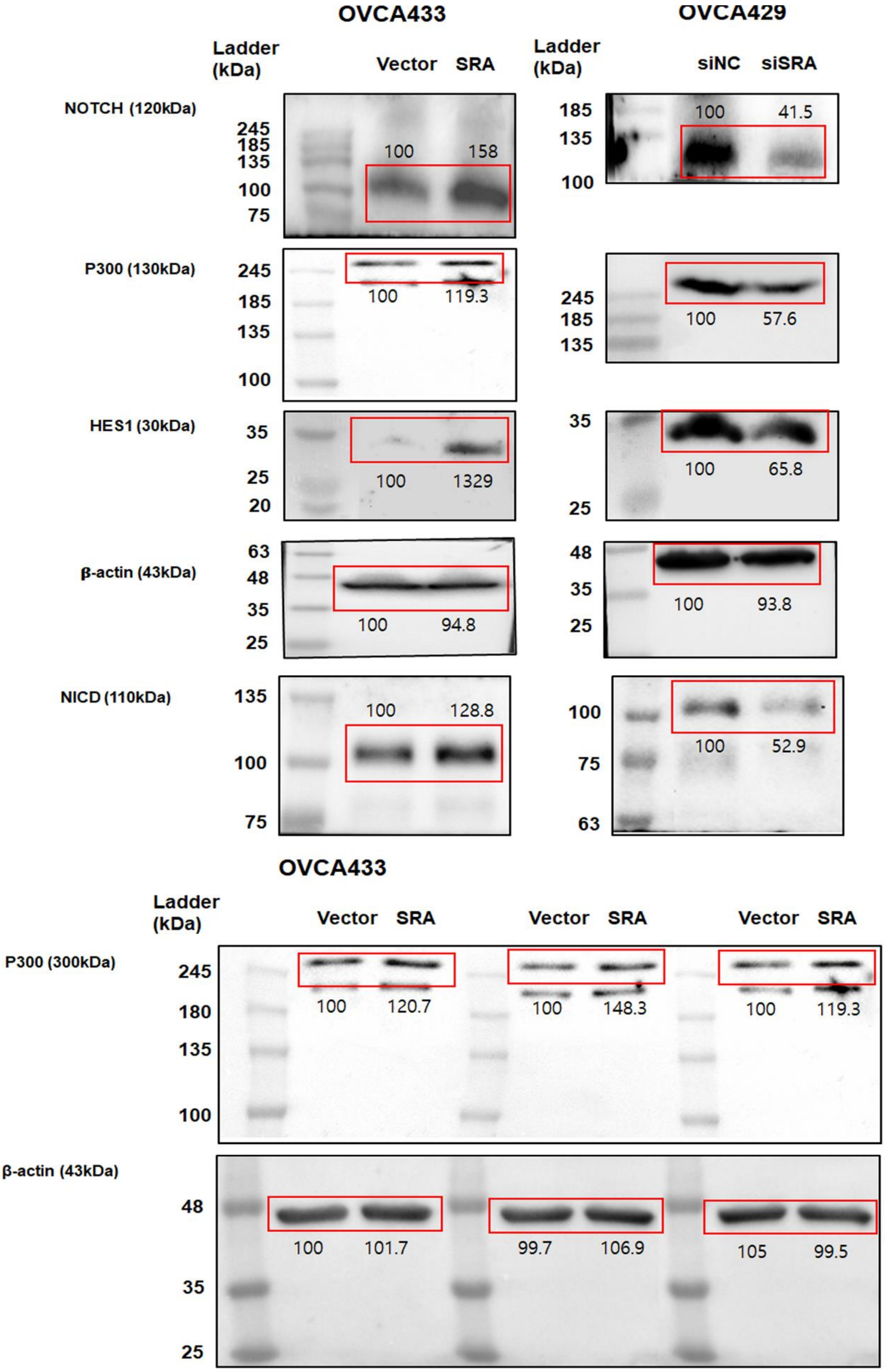

Figure S1. The Whole Western Blot for Figure 4.

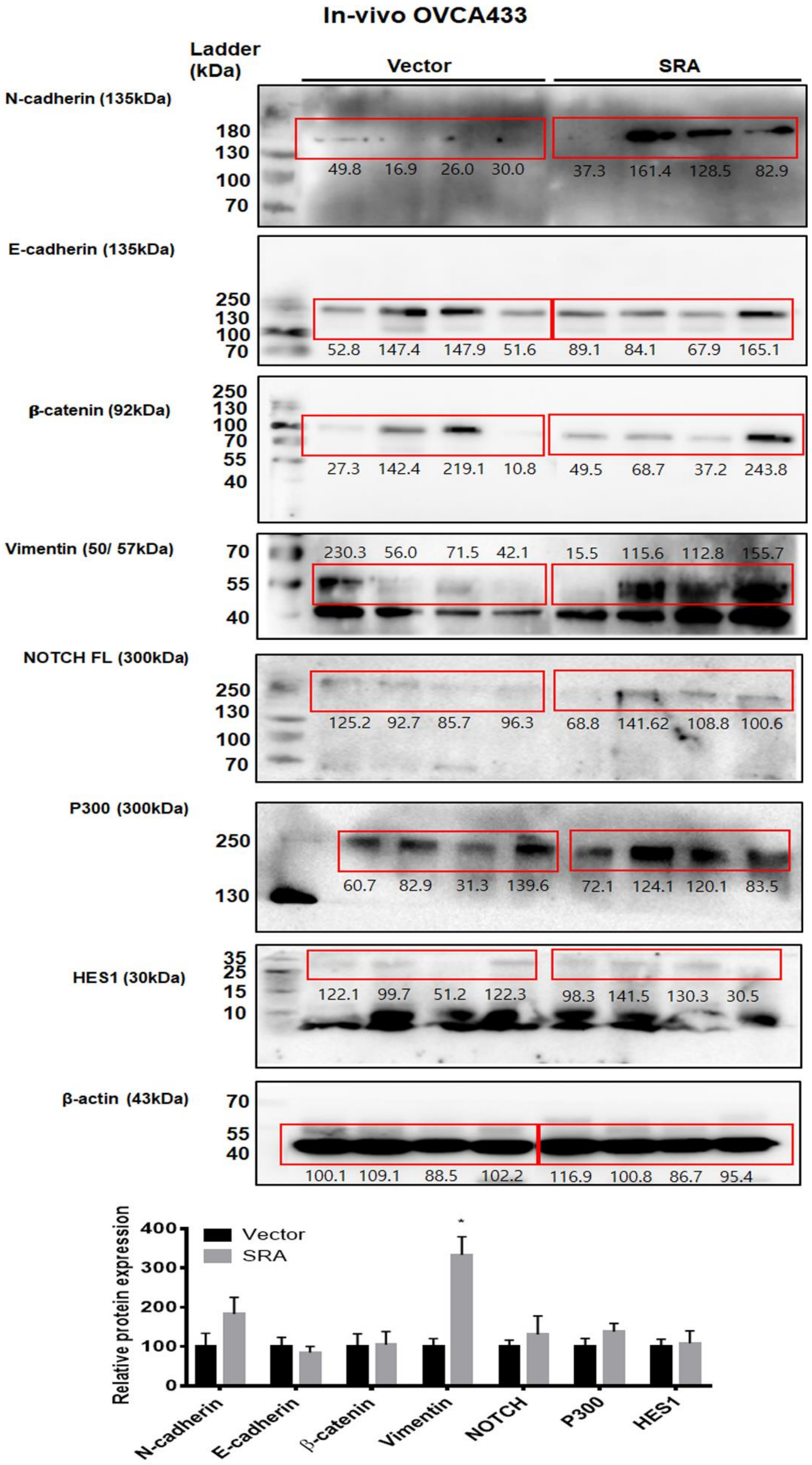

Figure S2. The Whole Western Blot for Figure 5.
